# Epigenetic Regulation in Kidney Transplantation

**DOI:** 10.3389/fimmu.2022.861498

**Published:** 2022-04-08

**Authors:** Xiaohong Xiang, Jiefu Zhu, Guie Dong, Zheng Dong

**Affiliations:** ^1^ Department of Nephrology, Hunan Key Laboratory of Kidney Disease and Blood Purification, The Second Xiangya Hospital of Central South University, Changsha, China; ^2^ Department of Cellular Biology and Anatomy, Medical College of Georgia at Augusta University and Charlie Norwood Veteran Affairs (VA) Medical Center, Augusta, GA, United States; ^3^ Department of Critical Care Medicine, The Second Xiangya Hospital, Central South University, Changsha, China; ^4^ Center of Nephrology and Dialysis, Transplantation, Renmin Hospital of Wuhan University, Wuhan, China

**Keywords:** kidney transplantation, epigenetic regulation, DNA methylation, acetylation, non-coding RNAs

## Abstract

Kidney transplantation is a standard care for end stage renal disease, but it is also associated with a complex pathogenesis including ischemia-reperfusion injury, inflammation, and development of fibrosis. Over the past decade, accumulating evidence has suggested a role of epigenetic regulation in kidney transplantation, involving DNA methylation, histone modification, and various kinds of non-coding RNAs. Here, we analyze these recent studies supporting the role of epigenetic regulation in different pathological processes of kidney transplantation, *i.e.*, ischemia-reperfusion injury, acute rejection, and chronic graft pathologies including renal interstitial fibrosis. Further investigation of epigenetic alterations, their pathological roles and underlying mechanisms in kidney transplantation may lead to new strategies for the discovery of novel diagnostic biomarkers and therapeutic interventions.

## Introduction

End stage renal disease (ESRD), as a consequence of diabetes, hypertension, obstructive nephropathy and chronic glomerulonephritis, has been increasing in the current aging society (1). Kidney transplantation (KT) is a preferred renal replacement therapy of ESRD for its advantage in higher survival rate and better quality of life (2). However, the donor shortage or long waiting list before KT, and poor transplant outcome or graft function loss after KT are two major concerns (3). Hence, it is important to clarify the pathophysiology and underlying mechanism of renal problems following KT for expanding the donor pool as well as improving the outcome of graft.

Kidney transplantation involves a series of pathophysiological changes from donor to recipient. First, ischemia-reperfusion injury (IRI) is the early event since grafts experience cold ischemia during cold storage, warm ischemia during cardiac arrest and surgery, and reperfusion injury right after graft revascularization (4). IRI is an independent risk factor for delayed graft function (DGF), which is defined as failure of transplanted kidney to function immediately and need dialysis for the first week post-transplantation (5). Moreover, graft rejection is a major obstacle of renal graft survival and it can be divided to acute rejection and chronic rejection. Acute rejection (AR) mostly happens within the first 3 months post-transplantation and is classified into acute T-cell mediated rejection (ACR) and acute antibody mediated rejection (AMR) (6). With the advancement of immunosuppressive drug, the incidence of acute rejection has been significantly reduced, but it still threatens 7.9% of transplanted patients (7). In contrast, chronic rejection that occurs months or years after kidney transplantation is the most prevalent cause of renal graft dysfunction currently (8). Chronic rejection or chronic allograft dysfunction (CAD) is a multifactorial process which involves immunological and non-immunological mechanism of kidney injury. Immune factors include acute rejection, poor histocompatibility, immunosuppressive agent insufficiency, *etc.*, while nonimmune risk factors mainly include poor donor quality, IRI, nephrotoxicity, hypertension, infection (9). Progressive interstitial fibrosis/tubular atrophy (IF/TA) is a remarkable pathological characteristic of CAD (10) but its underlying mechanism has not been fully clarified.

**Figure 1:**
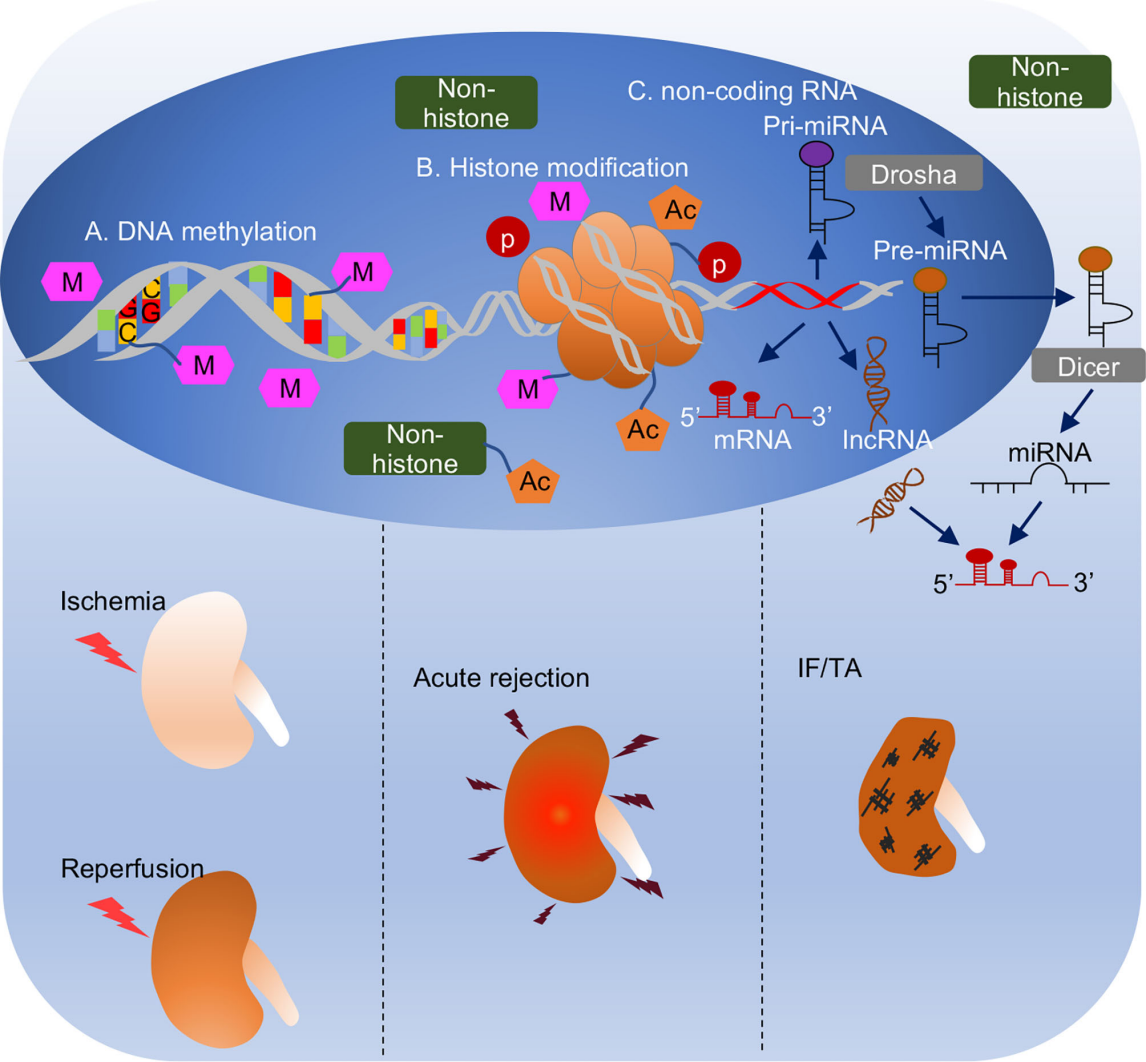
Overview of epigenetic regulation in different phases of kidney transplantation. **(A)** DNA methylation. DNA methylation is covalent binding of a methyl group to cytosine residues in CpG dinucleotide. DNA methylation generally correlates with transcriptional depression. **(B)** Histone modification. Two copies of core histones H2A, H2B, H3 and H4 assembled histone octamer, which is wrapped with DNA strand to form the basic unit of eukaryotic chromatin, nucleosome. Histones are accessible to be modified by acetylation, methylation, or phosphorylation, etc. Also, non-histones like transcriptional factors, transcriptional coactivators, or nucleus receptors can also be acetylated to modulate biological process. **(C)** Non-coding RNA is a kind of RNA that transcribed from DNA but not translated to proteins. It consists of miRNA with 21-23 nucleotides in length and lncRNA with over 200 nucleotides in length. MicroRNA is generated from pri-miRNA and pre-miRNA sequentially by Drosha enzyme in nucleus and Dicer enzyme in cytosol. Non-coding RNAs complementarily pair with mRNAs to regulate their activities (generally repression or degradation). Epigenetic mechanisms play important roles in different pathological processes in kidney transplantation, i.e., ischemia reperfusion injury when graft procured from donor to transplant into recipient, acute rejection usually happens within 3 months after transplantation, and interstitial fibrosis and tubular atrophy, a histological characteristic of chronic allograft dysfunction mainly caused 1-year post-transplantation.

In contrast to genetic mechanism, epigenetic regulation is broadly defined as an inheritable change resulting in gene expression without alteration of DNA sequence (11). Epigenetic regulation mainly consists of DNA methylation, histone modification, and non-coding RNAs, and it is one of the most rapidly progressing fields in elucidating kidney pathophysiology (12). The study of epigenetic regulation in kidney transplantation has received a lot of interest in the past decade, as reviewed in 2016 (13). It has been acknowledged that epigenetic regulation takes an important part in kidney transplantation related pathological events, from ischemia-reperfusion injury in the early phase (12, 14), immune response (15), to the development and progression of IF/TA in the late phase (16). For DNA methylation and histone modification, there were limited studies evaluating their roles in kidney transplantation in the past, but it has flourished since new characterization of a human DNA methylome and new histone modification were unveiled in the last 5 years. For non-coding RNAs, microRNAs have been studied for a long time, and the most recent advances mainly focus on their potential as non-invasive biomarker candidates in biological fluid (plasma or urine) or exosomes for their stability and resistance against cleavage by RNase (17). In the current review, we summarize these findings with a focus on DNA methylation, histone modification and non-coding RNAs in different phases of kidney transplantation (**Figure 1**).

## DNA Methylation in Kidney Transplantation

**Figure 2:**
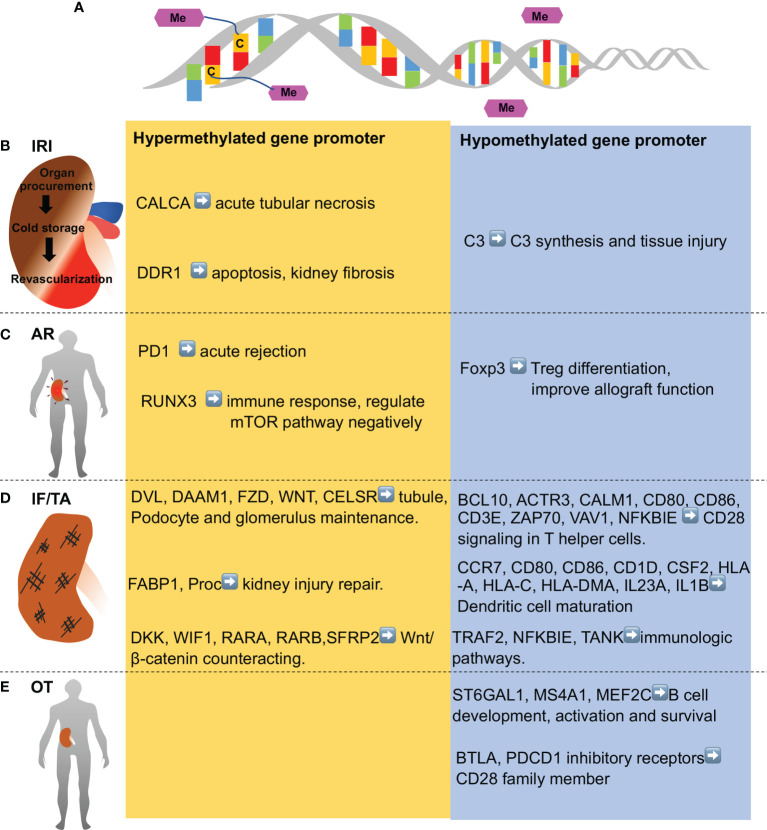
DNA methylation in various stages of kidney transplantation. **(A)** DNA methylation entails addition of methyl group to cytosine residues to form 5-methylcytosine in DNA.DNA demethylation is removal of a methyl group from cytosines. **(B)** Ischemia-reperfusion injury (IRI) occurs in kidney transplantation during organ procurement, cold storage, surgery and revascularization sequentially, and is associated with hypomethylation of some genes (e.g., C3 complement gene) and hypermethylation of others (CALCA, anti-apoptotic genes, and anti-fibrotic genes like DDR1). **(C)** Acute rejection (AR) shortly after kidney transplantation is associated with demethylation of Foxp3 gene and Treg fortification in immune tolerance patients, while PD1 and several genes in T cell receptor pathway and mTOR pathway (e.g., RUNX3) are hypermethylated. **(D)** Chronic rejection is characterized by interstitial fibrosis and tubular atrophy (IF/TA), which is associated with hypomethylation of the genes in inflammation and immune activation, whereas hypermethylation of the genes involved in kidney repair. **(E)** Operational tolerance (OT) is a condition of stable and acceptable graft function without the need of immunosuppressive drug. OT is associated with the hypomethylation of gene in B cell development, activation, and survival. Genes in CD28 family members are also hypomethylated in OT patients.

DNA methylation is the adding of a methyl group to cytosines in DNA resulting in the formation of 5-methylcystosine (5mC). DNA methylation mainly occurs in CpG island, where a high density of CpG dinucleotide exists. CpG island generally locates in the promoter and exon regions and its methylation mainly correlates with transcriptional repression (**Figure 2**) (18). DNA methylation is catalyzed by DNA methyltransferase, including DNMT1, DNMT3a and DNMT3b. In contrast, demethylation could be achieved either passively during cell division or actively *via* DNA methylation eraser enzymes (19).

### Ischemia-Reperfusion Injury and Delayed Graft Function

IRI is inevitable for kidney transplantation from deceased donors to recipients (20) and plays a pivotal role in occurrence of DGF, which negatively affects long term function of the transplant (21). DNA methylation has been reported to be implicated in renal IRI (14). In 2006, Pratt and colleagues demonstrated the demethylation at the C3 complement gene promoter in rat kidney with 24 hours of cold ischemia and a 2 hours of reperfusion in an isolated *ex-vivo* circuit, which led to local C3 synthesis and tissue injury (22). Their follow-up study showed that this demethylation of C3 promoter influenced pathological change in chronic allograft nephropathy (23). In contrast, Mehta et al. demonstrated the hypermethylation of CALCA (Calcitonin Related Polypeptide Alpha) gene in urine of KT patients with biopsy-proven acute tubular necrosis, suggesting the biomarker potential of the hypermethylation (24). In 2018, Heylen et al. (25) took advantage of microarray to analyze paired pre- and post-ischemic biopsies of brain-dead transplants in a longitude cohort to find out an overall increase in methylation after ischemia. Further cross-sectional cohort genome-wide DNA methylation analysis showed a cold ischemia time-dependent hypermethylation in majority of methylated CpG sites. Functionally, IRI-induced hypermethylation predominantly involved in suppression of anti-apoptotic and anti-fibrotic genes and correlated with chronic allograft injury. For example, the highest increase in methylation is in the DDR1 promoter, which is known to be involved in apoptosis and kidney fibrosis. These findings indicate that DNA methylation represents a promising therapeutic target for preventing cold ischemia-associate acute injury and chronic allograft injury.

### Acute T Cell-Mediated Rejection and Acute Antibody-Mediated Rejection

Acute rejection is recognized as one of the most important cause of graft loss and it is composed of aforementioned ACR and AMR (9). During ACR, mononuclear cells like CD4^+^ or CD8^+^ T cells accumulate in interstitium, along with cytokines or chemokines such as interferon γ (IFN γ), tumor necrosis factor β (TNF β), TNF α, *etc. *(10). CD4^+^Foxp3^+^ regulatory T (Treg) cells are essential for the maintenance of immune tolerance through suppressing excessive immune response (26) and their characteristic transcriptional factor Foxp3 is regulated epigenetically to modulate immune homeostasis (27, 28). Bestard et al. reported that demethylation of Foxp3 is associated with intra-graft higher expression of Treg cells and a favorable long-term allograft outcome in subclinical rejection patients (histologic evidence of rejection exists in biopsy but lack of clinical kidney dysfunction) (29). In contrast, Boer et al. reported that hypermethylation of the immune inhibitory receptor programmed death 1 (PD1) in CD27^-^ memory CD8^+^ T cell correlates positively with acute rejection at 3 months (30). Therefore, different genes may be subjected to the regulation by methylation in different T cells affecting the immune response and outcome of kidney transplantation. In 2020, Zhu et al. analyzed DNA methylation in peripheral blood mononuclear cells (PBMC) of patients with or without AR-induced allograft dysfunction and demonstrated that hypermethylated genes are enriched in T cells and mechanistic target of rapamycin signaling (mTOR) pathways in AR-related graft dysfunction. Further work using mice AR model of renal transplantation showed that the DNMT inhibitor decitabine could ameliorate kidney allograft inflammatory injury *via* demethylating and enhancing the negative regulators of mTOR signaling (31). These results indicate that inhibition of DNA methylation may provide therapeutic effects against acute renal allograft rejection.

### Chronic Rejection and Interstitial Fibrosis/Tubular Atrophy

IF/TA is a main signature of late allograft dysfunction and the mechanism leading to IF/TA includes inflammation, renal fibroblast activation, and deposition of extracellular matrix (32). Several lines of evidence indicate that DNA methylation plays a role in development and progression of IF/TA in renal allograft. In 2017, Bontha and colleagues firstly used integrative multi-omics approach (methylation arrays, gene expression arrays and miRNA arrays) to evaluate the overall distribution of epigenetic modifications across genes and showed their relationship with renal graft IF/TA, function and long-term outcome. By comparing the biopsies of IF/TA and non-fibrosis/atrophy (NFA) at 24 months post-transplantation, they observed that enrichment of hypomethylated CpGs sites correlated with inflammation in IF/TA biopsies, whereas hypermethylation corresponded to the inhibition of kidney repair. This study also showed that hypomethylation could also modulate immune genes expression by regulating miRNAs (33). As mentioned above, Heylen et al. suggested that ischemic insult caused general hypermethylation and predicted CAD 1 year after transplant (25). Their further study unveiled the mechanism underlying aging donor related kidney allograft dysfunction. Donor aging associated differentially methylated regions were frequently located in the genes involved in the Wnt/β-catenin signaling pathway, which affect glomerulosclerosis and interstitial fibrosis as well as graft function at one year after transplantation (34). In addition, Rodriguez et al. performed genome-wide analysis of DNA methylation in PBMCs from KT patients with chronic rejection or operational tolerance (OT), a condition of stable graft acceptance without the need for immunosuppression therapy. They found that OT is associated with demethylation in genes involved in immune function like T, B cell activation and Th17 differentiation, while chronic rejection is related to demethylation in intracellular signaling and ubiquitination pathways (35). The question is arising how epigenetic mechanisms can affect operational tolerance. Previous transcriptomic analysis showed that OT was associated with B cell profile (enrichment of B cells especially naïve and transitional B cells which can produce IL10 and inhibit CD4+ T cells) (36, 37). Consistently, the DNA methylation profile of OT patient showed demethylation of CD20 encoding genes to result in survival of transitional B cells and expansion of these populations (35). Operational tolerance is also related to poor Th17 response and reduced TCR signaling (38, 39). Although partially, the authors observed some molecular pathways associated with Th17 functions. For example, they found demethylation of PD1 and BTLA and the genes associated with negative regulation of ERK and NF-κB, which led to damage of T-cell activation and Th17 response (35).

A most recent study compared epigenome-wide methylation modification between healthy patients and renal failure replacement therapy patient (dialysis and kidney transplantation) at baseline (right before renal failure replacement therapy) and over 12 months treatment, they found that uremic milieu drives genome-wide methylation changes but partially reversed with kidney failure replacement therapy. However, 413 CpG sites remained differentially methylated at follow-up in renal replacement therapy, which merit further investigation (40). These observations suggest a role of DNA methylation in chronic inflammation, IF/TA and chronic allograft dysfunction in kidney transplantation. Future investigation needs to pinpoint the specific genes and related mechanism underlying their pathogenic functions.

## Histone Modification in Kidney Transplantation

Histones are basic structural proteins in eukaryotic chromosomes, where they are wrapped with DNA to form nucleosome. Core histones (H2A, H2B, H3 and H4) in nucleosome are abundant in lysine and arginine residues, which are easily accessible for modification (41). Histone modification includes various forms like histone acetylation, methylation, phosphorylation, ubiquitylation, SUMOylation, citrullination, biotinylation, crotonylation and adenosine diphosphate (ADP) ribosylation, *etc.* (12). Acetylation is the most common modification in histones and is the focus of most studies in kidney pathogenesis, while only a little evidence is available for other histone modifications especially in kidney transplantation (42). Histone acetylation entails the addition of acetyl group to lysine residues, and this addition facilitates relaxation of nucleosome structure and transcriptional activation in general. Conversely, histone deacetylation refers to removal of acetyl group to result in transcriptional repression by allowing chromatin compaction. Acetylation and deacetylation are in dynamic equilibrium modulated by histone acetyltransferases (HATs) and histone deacetylases (HDACs) (42). HATs consist of 3 major families: GCN5 (includes GCN5 and PCAF), p300 (contains CBP and p300) and MYST (includes TIP60 and other 4 enzymes). HDACs are classified to class I (HDAC 1, 2, 3 and 8), class II (HDAC 4, 5, 6, 7, 9 and 10), class III Sirtuin family (Sirt1- Sirt7) and class IV (HDAC11) (43). HATs and HDACs can also modulate de/acetylation in non-histone proteins, so they are also termed as lysine acetyltransferases (KATs) and lysine deacetylase (KDACs), respectively (44). Non-histone acetylation is an important part of acetylome in mammalian cells and its involvement in kidney transplantation was raised in recent years.

### Histone Acetylation in Kidney Transplantation

In 2008, Marumo et al. firstly demonstrated that renal ischemia in mice induced a transient decrease in histone acetylation in proximal tubules. During reperfusion, HDAC5 was downregulated in parallel with the recovery of histone acetylation. Downregulation of HDAC5 was associated with the acetylation and expression of BMP7, which contributed to kidney repair and regeneration (45). In 2015, Levine et al. reported that the pan-HDAC inhibitor trichostatin (TSA) and class I specific inhibitor MS-275 ameliorated renal IRI in acute phase and diminished fibrosis formation in the long term. They also conducted renal syngeneic transplantation with prolonged cold ischemia in mice to figure out the anti-fibrotic effect of TSA (46). To elucidate the specific role of individual HDAC isoforms, their most recent study showed that global or renal tubule conditional (PAX8^cre^) inducible ablation of HDAC2 (but not HDAC1) protected against IRI-induced renal injury and formation of fibrosis. Likewise, in cold storage/transplantation model, mice received HDAC2^-/-^ isografts showed superior survival compared with recipients of WT isografts and had the trend of long-term functional protection and less fibrosis. These results support a pathogenic role of HDAC2 in renal IRI during kidney transplantation (47). Our latest study found that HDAC3 specific inhibitor RGFP966 diminished cold storage/transplantation injury and improved renal allograft function in mice kidney transplantation. This inhibitor and HDAC3 knockdown also protected against cold storage/rewarming injury in rat kidney proximal epithelial cells, indicating HDAC3 also takes part in IRI of kidney transplantation (48). In the sirtuin family, SIRT1 (49, 50), SIRT3 (51) and SIRT5 (52) were reported to reduce kidney IRI mainly through mitochondrial mechanisms (mitochondria dynamic and biogenesis). In view of the crucial role of mitochondria in warm and cold renal IRI in kidney transplantation (53, 54), agonists or activators of SIRT1, SIRT3, and SIRT5 may mitigate injury and improve graft function in kidney transplantation. There are considerable studies implicating HDACs in kidney fibrosis (55–57), but very limited is known about the involvement of HDACs in IF/TA in renal transplantation. In aforementioned reports by Levine and colleagues, TSA not only ameliorated acute ischemia-reperfusion injury, but also reduced fibrosis substantially in kidney syngeneic transplantation with prolonged cold storage (46). Specifically, HDAC2 knockout grafts showed the trend of less fibrosis than WT grafts (47). Zou et al. established F344-Lewis rat CAD model to find that Sirt1 (58) and Sirt3 (59) were both decreased at 3 months after transplantation, and their expression negatively correlated with renal failure, inflammatory chemokines expression and interstitial fibrosis severity, suggesting that sirt1 and sirt3 may serve an important protective role in the early stage of CAD. However, the underlying mechanisms and the roles of other HDACs in chronic renal rejection and associated fibrogenesis remains unclear. Obviously, further research in this area will provide new insights into allograft function loss and may lead to new therapeutic target.

### Non-Histone Acetylation in Kidney Transplantation

**Figure 3:**
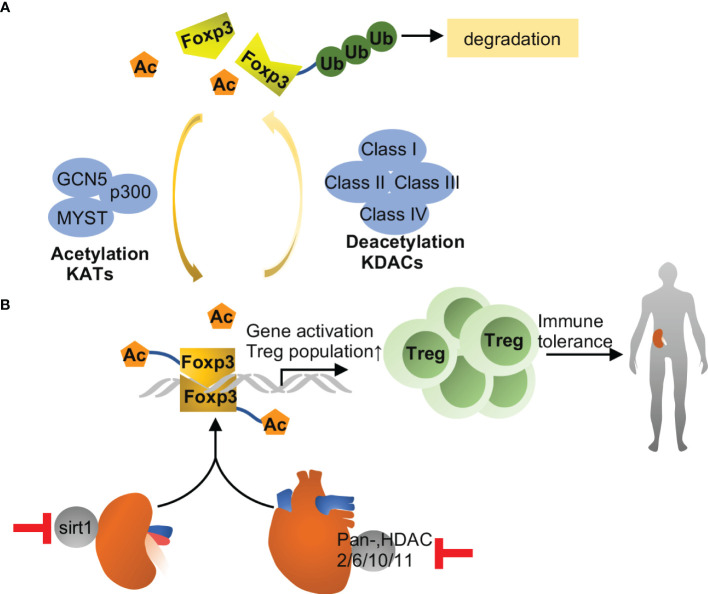
Foxp3 acetylation in transplantation. **(A)** Foxp3 acetylation is the addition of acetyl groups to lysine residues in Foxp3, which is catalyzed by lysine acetyltransferases (KATs), including GCN5, p300 and MYST. Acetylation promotes Foxp3 dimerization, DNA binding and transcriptional activity, and expansion of the Treg population, and associated with immune tolerance eventually. Conversely, Foxp3 deacetylation is catalyzed by lysine deacetylase, including class I, II, III and IV family members. Upon deacetylation, Foxp3 is prevented from dimerization and prone to poly-ubiquitination and proteasomal degradation. **(B)** Inhibition of Sirt1 improves immune tolerance and survival during kidney transplantation in mice, while pharmacological inhibition of other HDACs may improve allograft tolerance in heart. The effects are likely related to increased acetylation of Foxp3 and consequent expansion of Tregs.

Non-histone acetylation is complicated with diverse human diseases and key cellular processes. Mostly extensively studied proteins that can be acetylated includes p53, tubulin, p65, heat shock protein-90, *etc.*, with more and more non-histone proteins being recognized nowadays (44). Foxp3 acetylation is an important modulatory mechanism for immune response in solid organ transplantation (60). Acetylated Foxp3 is more stable than its non-acetylated form, because it prevents ubiquitination at target lysine residues and, in turn, proteasomal degradation (**Figure 3**). Acetylation also improves the ability of Foxp3 binding to chromatin and augments its function as a transcriptional regulator in Treg cells (61). In 2016, Levine et al. (62) reported that inhibition of SIRT1 led to the stabilization of the Treg phenotype by increasing Foxp3 acetylation resulting in better renal allograft survival and function in mice. Specifically, in a mouse model of renal allograft transplantation, CD4-Cre directed ablation of sirtuin-1 as well as a sirtuin-1 specific inhibitor (EX-527) improved the survival and kidney function in recipient mice. It is worth noting that EX-527 at higher doses did not improve renal allograft function but trend to pose inferior effects, suggesting that SIRT1 may act protectively on non-T cells (49, 50, 63). These studies indicate that the time window and dose of Sirt1 inhibitors used in targeting immune response merit careful consideration. Similarly, it is plausible to speculate that inhibition of Zn-dependent HDACs has the potential to fortify immune tolerance by increasing Foxp3 acetylation in Tregs. In this regard, Levine and colleagues published a series of impressive studies using the model of cardiac allograft transplantation. They initially reported that the pan-HDAC inhibitor TSA in conjunction with Rapamycin induced permanent, Treg-dependent cardiac allograft survival and donor-specific graft tolerance (64). Then, they found that recipient mice receiving HDAC6-knockout Treg showed better cardiac allograft survival than those receiving WT Tregs (65). Moreover, Treg-specific deletion of HDAC2 (66), HDAC10 (67) and HDAC11 (68) promoted Treg function and prolonged cardiac allograft survival significantly. Despite these remarkable observations in cardiac allografts, there is no report about HDAC inhibitors in augmenting immune tolerance in kidney transplantation. It is still a long way for clinical use of HDAC inhibitors for the difficulty to have specific, non-toxic inhibitors and the complexity of biological effects of HDACs.

### Other Histone Modification in Kidney Transplantation

In addition to acetylation, there is evidence supporting the role of methylation and phosphorylation of histone in renal IRI or kidney transplantation. Histone methylation is the addition of methyl group to lysine or arginine of core histones, which is mediated by histone methyltransferase (HMT) (69). Different from histone acetylation, histone methylation may be associated with gene transcriptional permission or repression. The effect depends on the amino acid residue methylated and the extent of modification (mono-, di-, and tri-) (70). For example, H3K4 methylation is associated with activation of transcription, while methylation of H3K27 and H3K9 results in transcriptional silencing (71). Naito et al. reported that in unilateral IRI mice, H3K4m3, H3K9ac and H2A.Z variant of HMG CoA reductase gene were increased at their promoters and exons. These permissive modifications allow for tubular cholesterol accumulation and renal protection (72). In contrast, another study showed that unilateral IRI in rat induced the recruitment of H3K4m2 to the promoter of TGF-β, which is consistent with TGF-β transcription and kidney injury (73). Matrix chromatin immunoprecipitation showed that renal IRI induced permissive histone marks (H3K9Ac, H3K18Ac, H3K27Ac, H4K5/8/12/16Ac, H3K4m3, H3K4m2 and H3S10ph) at the proinflammatory gene TNF-α, whereas repressive histone mark (H3K27m3) at the gene was decreased (74). In terms of histone methyltransferase, enhancer of zeste homolog 2 (EZH2) (75, 76) and G9a (77) catalyze methylation of H3K27 and H3K9/H3K27, respectively. Their inhibitors were reported to ameliorate renal IRI. What’s more, EZH2 inhibitor suppressed immune response of alloreactive T cell and inflammatory cytokine activation, resulting in attenuation of acute allograft rejection in rat kidney transplantation (78). Zhang et al. used chromatin immunoprecipitation with DNA microarray (CHIP-chip) to determine H3K4 methylation of monocytes in acute rejection or non-AR rats. They detected significant difference in H3K4m3 levels in 141 probes, but their correlation with AR after kidney transplantation needs further clarification (79).

Histone phosphorylation involves phosphorylation of serine, threonine and tyrosine residues on histones mediated by protein kinases and phosphatases (80). The best-known function of histone phosphorylation is in DNA damage response, where H2AX variant is phosphorylated on serine 139 to mediate DNA double strand break repair (81). Besides, histone phosphorylation can also function in transcriptional regulation and chromatin compaction, the latter is associated with mitosis/meiosis and apoptosis (80). Our study in 2014 firstly demonstrated the induction of phosphorylation of H2AX and its kinase ATM in renal IRI, indicating involvement of DNA damage response in this disease (82). Of note, histone modification by these mechanisms is a largely untapped area to explore in kidney transplantation.

## Non-Coding RNAs in Kidney Transplantation

Non-coding RNAs (ncRNAs) include a diverse family of RNAs that are not translated to proteins. In particular, microRNA (miRNA) and long ncRNA have been intensively investigated in recent years (83). MiRNA is a single-stranded RNA of 21-23 nucleotide in length and complementarily pairs with its target mRNA transcripts to induce mRNA degradation or prevent their translation. The production of mature or functional miRNAs involves sequential cleavage by two RNase, Drosha in the nucleus and Dicer in the cytosol (84). Long ncRNA (LncRNA) is a type of transcript over 200 nucleotides in length that functions both transcriptionally and post-transcriptionally by interacting with DNA, RNA, and proteins. It can also bind to microRNAs to titrate them away from their targets (85). In addition, circular RNA (circRNA) is a kind of newly recognized long ncRNA which forms a covalently closed loop in topology (86). Over the past decade, miRNAs have been extensively studied in kidney transplantation as reviewed in 2019 (17). LncRNAs have emerged as important epigenetic regulators in kidney transplantation and might be potential biomarkers according to recent reports. Here, we mainly provide updated information of miRNAs (**Table 1**) and an overview of emerging lncRNAs in kidney transplantation (**Table 2**).

**Table 1:** MicroRNAs in kidney transplantation.

Process	Study	Design	Sample	MicroRNAs	Expression	Function
**IRI**	Lee et al. (87)	Post-KT (n=5) Pre-KT (n=5)	Blood	miR-let-7a-3p, -143-3p, -214-3p	↑	unknown
				Let-7d-3p, let-7d-5p, miR-1246, -1260b, -1290, -130b-3p	↓	unknown
IRI/DGF*	Khalid et al. (88)	DGF (n=33) IGF (n=33)	Urine	miR-9, -10a, -21, -29a, -221, -429	↑	Predict DGF
**IRI/DGF**	Li et al. (89)	abnormal Cr* (n=59) Normal Cr (n=45)	PBMC	miR-142-5p, -142-3p, -223	↑	Predict allograft dysfunction
				miR-10b	↓	Predict allograft dysfunction
**IRI/DGF**	Wang et al. (90)	DGF (n=4) IGF (n=5)	Exosomes	miR-33a-5p_R-1, -98-5p, -151a-5p	↑	Predict DGF
AR*	Pang et al. (91)	Mice allogenic KT model	imDECs*	miR-682		Suppress AR
**AR**	Liu et al. (92)	Mice allogenic KT model	biopsy	miR-15b		Suppress AR
**AR**	Liang et al. (93)	Rat allogenic KT model	plasma	miR-155	↑	Predict AR
**AR**	Gielis et al. (94)	AR (n=15) control (n=16)	Urine	miR-155-5p	↑	Predict AR
				miR-615-3p	↓	Predict AR
ACR*	Quintairos et al. (95)	AR (n=8) non-AR (n=50)	urine	miR-155-5p	↑	Predict AR and monitor therapy
**AR**	Alfaro et al. (96)	AR (n=5) non-AR (n=10)	PB leukocytes	miR-150-5p	↓	Promote immune response
						
**AR**	Freitas et al. (97)	23 kidney recipients	Exosomes	miR-155-5p	↑	Monitor therapy and graft function
				miR-223-3p, 1228-3p	↓	Monitor therapy and graft function
AMR*	Tinel et al. (98)	ABMR vs Non-ABMR	Biopsy	miR-142-3p, -150-5p, -155-5p, -222-3p, -223-3p	↑	Correlate with MVI*, AMR
				miR-139-5p	↓	Correlate with MVI*, AMR
**AMR**	Kuscu et al. (99)	TG* (n=34) control (n=19)	Plasma	miR-1224-5p, -4508, -320, -378a	↓	Promote immune response
OT*	Cabral et al. (100)	OT (n=8) CR (n=5)	Plasma	miR-885-5p miR-331-3p, -27a-5p	↑	graft survival unknown
				miR-1233-3p, -572, -638, -1260a	↓	unknown
**IF/TA**	Xiong et al. (101)	IFTA (n=14) Health (n=8)	Biopsy	miR-378	↓	Reduce IF/TA
CAD*	Chen et al. (102)	53 kidney recipients	Exosomes	miR-21, -210, -4639	↑	Predict CAD
IF/TA*	Gniewkiewicz et al. (103)	IFTA high grade* (n=14) IFTA low grade* (n=17)	Urine	miR-21	↑	Predict IF/TA, graft dysfunction.
**IF/TA**	Saejong et al. (104)	IFTA high grade (n=21) IFTA low grade (n=15)	plasma exosomes	miR-21	↑	Predict IF/TA, graft dysfunction.

IRI/DGF*, ischemia reperfusion injury/delayed graft function; Cr*, creatinine; AR*,acute rejection; imDECs*, immature dendritic cells-derived exosomes; ACR*, acute T-cell mediated rejection; AMR*,acute antibody mediated rejection; MVI*, microvascular inflammation; TG*, transplant glomerulopathy; OT*, operational tolerance; CAD*,chronic allograft dysfunction; IF/TA*, interstitial fibrosis/tubular atrophy; IFTA high grade*, grade II (25-50% IFTA) and III (≥50%); IFTA low grade, grade I (0-25% IFTA). ↑, up-regulated; ↓, down-regulated.

**Table 2:** LncRNAs in kidney transplantation.

Process	Study	Design	Sample	LncRNAs	Expression	Function
**IRI/DGF**	Pang et al. (105)	Mice syngeneic KT model	Biopsy	MEG3	↑	Promote DGF
**IRI/DGF**	Nagarajah et al. (106)	DGF (n=22) IGF (n=107)	Blood	MGAT3-AS1	↓	Predict DGF
**IRI/AR**	Zou et al. (107)	Rat allogenic KT model	biopsy	PRINS	↑	Unknown
**AR**	Dai et al. (108)	AR (n=3) healthy (n=3)	Biopsy	32 dysregulated lncRNAs		Unknown
**AR**	Dai et al. (109)	AR (n=3) healthy (n=3)	Biopsy	uc010ftb AK129917	↑	Unknown
				uc003wbj, uc001fty AF113674	↓	Unknown
**ACR**	Qiu et al. (110)	AR (n=72) control (n=36)	Biopsy	LncRNA-ATB	↑	Predict AR, graft dysfunction
**ACR**	Lorenzen et al. (111)	AR (n=62) control (n=31)	Urine	RP11-354P17.15-001	↑	Predict AR, graft dysfunction
**AR**	Ge et al. (112)	AR (n=150) stable (n=150)	Plasma	AF264622 AB209021	↑ ↓	Predict AR Predict AR
**AR**	Nafar et al. (113)	AR (n=29) stable (n=32)	PB	FAS-AS1	↑	Unknown
**ACR**	Groeneweg et al. (114)	AR (n=15) stable (n=32)	Plasma	LNC-EPHA6	↑	Predict AR
**AR**	Wang et al. (115)	Rat allogenic KT model	BMSC-sEVs	Loc108349490	↑	Alleviate AR
**AR**	Wu et al. (116)	Mice allogenic KT model	BMSC-Ex	DANCR	↑	Alleviate AR
**ACR**	Kölling et al. (117)	ACR (n=62), stable (n=31)	Urine	hsa_circ_0001334	↑	Predict AR, graft dysfunction
**CAD**	Xu et al. (118)	Bioinformatic analysis	Biopsy	AC126763.1, RP11-280K24.1, LINC01137, WASIR2, RP1-276N6.2, AD000684.2.	↑	Predict CAD

↑, up-regulated; ↓, down-regulated.

### Ischemia-Reperfusion Injury and Delayed Graft Function

In 2010, our laboratory generated a proximal tubule (PT) specific Dicer ablation mouse model and demonstrated the resistance of these mice to renal IRI, suggesting a pathogenic role of miRNAs (119). In the same study, our microarray analysis further identified the specific microRNAs that were up- or down- regulated in renal IRI (119). Lee et al. extracted peripheral blood from kidney transplant recipient right before transplant surgery and immediately after reperfusion, in the purpose of comparing the microRNA profile of non-AKI and AKI in kidney transplantation. They observed 3 upregulated and 6 downregulated miRNAs post-reperfusion. But their function in IRI of kidney transplantation need further clarification (87). In 2019, Khalid et al. used microarray analysis of “first-pass urine” sample (taken immediately post-transplant) from DGF and non-DGF patients and identified 6 upregulated microRNAs (miR-9, -10a, -21, -29a, -221, and -429) in DGF (88). Another independent cohort further verified the increase trend of the 6 candidate microRNAs in urine samples at varying time in the first week post-transplantation, with miR-21 being the most evident (88). This study suggested the biomarker potential of the urinary microRNAs for early prediction or diagnosis of DGF following kidney transplantation. Besides, miRNAs in peripheral blood have been suggested as promising non-invasive biomarkers. For example, upregulation of miR-142-5p, miR-142-3p and miR-223 and downregulation of miR-10b were detected in peripheral blood mononuclear cells (PBMC) at 3-4 weeks earlier than renal dysfunction shown by serum creatinine increase in human kidney transplantation patients. The elevations of miR-142-5p and miR-142-3p were also associated with later cystatin C increases. These results indicate that miRNAs in PBMC may be useful in predicting renal allograft dysfunction than conventional biomarkers, such as serum creatinine and cystatin C (89). Recently, exosomes, a type of extracellular vesicle carrying abundant contents including miRNAs, have attracted extensive interest (120). Wang et al. applied high-throughput sequencing to explore the miRNA expression profile in exosomes from peripheral blood of allograft recipients with or without DGF. They observed the up-regulation of hsa-miR-33a-5p_R-1, hsa-miR-98-5p, and hsa-miR-151a-5p in DGF recipients and demonstrated a positive correlation of hsa-miR-151a-5p with first-week renal function post-transplantation (90).

As for lncRNAs, Pang et al. examined a mouse syngeneic kidney transplantation model and demonstrated the upregulation of a specific LncRNA called maternally expressed gene 3 (MEG3) in the first few days of post-engraftment (105). Notably, silencing MEG3 resulted in the attenuation of kidney damage and dysfunction, suggesting an injurious function of MEG3 in this model. Mechanistically, MEG3 was shown to compete with miR-181b to induce TNF-a and related acute renal allograft injury (105). Nagarajah et al. collected blood samples from deceased donor kidney transplant with DGF or immediate graft function (IGF). In these samples, lower lncRNA MGAT3-AS1 was associated with DGF, indicating its predictive role in short-term outcome of transplantation (106). Zou et al. compared prolonged cold ischemia (16h) rat allogeneic kidney transplantation with short ischemia (2h) rat syngeneic or allogeneic transplantation. They found that lncRNA PRINS (Psoriasis susceptibility-related RNA gene induced by stress) was elevated in allograft after 16h of cold ischemia and correlated with acute tubule damage (107). However, in the absence of prolonged ischemia syngeneic transplantation group, it’s hard to discriminate whether the lncRNA PRINS play a role in AKI or acute rejection.

### Acute T Cell-Mediated Rejection and Acute Antibody-Mediated Rejection

Pang et al. found that injection of immature dendritic cells-derived exosomes to renal allograft mice promoted Treg cell differentiation and immune tolerance (91). The protective effect was shown to be mediated largely by miR-682 in the exosomes, supporting the beneficial effect of miR-682 in AR (91). In allogeneic renal transplantation mice, miR-15b mediated the protective effect of Bortezomib (a highly selective proteasome inhibitor) against AR through inhibiting T follicular helper (Tfh) cell proliferation and differentiation, suggesting a critical role of miR-15b in suppressing Tfh cell activity and related acute immune response (92). For biomarker application, Liang et al. established F344-Lewis rat AR model to demonstrate that plasma miR-155 was increased during AR and correlated with the severity of AR (93). Gielis et al. further showed that miR-155-5p and miR-615-3p in urine sediments, together with chemokine CXCL-9 in urine supernatant, may discriminate kidney transplant rejection from stable graft conditions, suggesting the combined use of urinary molecular biomarkers for the detection of rejection (94). Similarly, another study using logistic regression analysis showed that urinary miR-155-5p could identify patients with potential high risk of rejection at early stage of post-transplantation (95). Alfaro et al. showed that miR-150-5p was decreased in acute rejection kidney transplantation patients, the decrease could be related to activation and proliferation of the immune system’s cells (96). A pilot study showed that in the first 3 months post transplantation with tacrolimus therapy, urinary exosome derived miR-155-5p and miR-223-3p correlated with tacrolimus dose, miR-223-3p with serum creatinine, and miR-223-3p and miR-1228-3p with blood leukocytes, indicating that differentially expressed urinary exosome miRNAs might monitor tacrolimus therapy and renal graft function (97). In contrast to the leading role of ACR in kidney transplantation, AMR characterized by microvascular inflammation (MVI) is less studied. However, a recent study unraveled 6 differently expressed miRNAs between AMR biopsies and non-AMR. Integrative omics profiling of miRNAs and mRNAs and single-cell RNA sequencing revealed new pathways involved in MVI and AMR in different cell types, including endothelial cells, epithelial cells and immune cells (98). Moreover, as a histological hallmark of AMR, transplant glomerulopathy is associated with down-regulation of miRNAs like miR-1224-5p, -4508, -320, -378a. The downregulation of these miRNAs results in upregulation of their target genes and activation of T helper cells, dendritic cell maturation and Th1/Th2 pathways (99).

For LncRNAs, Dai and colleagues identified 5 transcriptional factors that were associated with 12 miRNAs and 32 lncRNAs in biopsies of 3 patients with AR (108). Their subsequent microarray analysis of lncRNA in AR biopsies revealed 5 differentially expressed lncRNAs (109). However, these studies are limited by small sample size and absence of assessing the predictive value of these lncRNAs. Qiu et al. (110) detected a higher level of lncRNA-ATB (lncRNA activated by transforming growth factor β) in ACR biopsies. LncRNA-ATB inversely correlated with miR-200c and acted as a sponge for miR-200c. In biological fluid, Lorenzen et al. conducted genome-wide analysis of RNA from urine of patients with ACR. They figured out a novel lncRNA named RP11-354P17.15-001 that significantly increased in ACR patients compared with healthy controls and normalized to control level after successful anti-rejection therapy. Moreover, RP11-354P17.15-001 was associated with a more severe decline in glomerular filtration rate at 1 year post-transplantation and it allowed detection of subclinical rejection patients that would have been missed by routine serum creatinine measurement (111). Genome-wide lncRNA analysis in circulating peripheral blood (PB) uncovered 23 deregulated lncRNAs which could discriminate AR from those without AR. Among them, AF264622 and AB209021 showed the best diagnostic capabilities (112). Nafar et al. showed that lncRNA FAS-AS1 was higher in rejection patients when compared with non-rejection ones in males but not in females, suggesting its putative role in the pathogenesis of renal transplant rejection (113). In addition, circulating plasma LNC-EPHA6 (a vascular injury related lncRNA) showed higher expression in ACR than that in stable patients (114). For extracellular vesicular lncRNAs, Wang et al. showed that bone marrow mesenchymal stem cell (BMSC)-derived small extracellular vesicles (sEVs) mitigated inflammation and renal dysfunction in a Sprague-Dawley (SD) to Wistar rat renal transplantation model, and the effect was mainly mediated by LncRNA Loc108349490 (115). Similarly, in a mouse renal allograft model, BMSC-derived exosomes (BMSC-Ex) implantation promoted Treg cell quantity and improved immune tolerance, which was mediated by lncRNA DANCR (differentiation antagonizing non-protein coding RNA) (116). CircRNAs exist abundantly in eukaryotic transcriptome and function mainly by sponging and sequestering miRNAs (86). A recent study analyzed the global circRNA expression pattern in urine of patient with ACR and their control transplants. It was found that hsa_circ_0001334 upregulation not only correlated with acute kidney rejection, but predicted loss of kidney function 1 year after transplantation (117).

### Chronic Rejection and Interstitial Fibrosis/Tubular Atrophy

To identify the molecular basis underlying operational tolerance (OT), Cabral et al. used low density array to reveal 3 higher level (miR-885-5p, -331-3p, -27a-5p) and 4 lower level of miRNAs (miR-1233-3p, -572, -638, -1260a) in serum of OT patients compared with that of chronic rejection patients (100). Gene set enrichment analysis indicated that these miRNAs target cell death regulation/survival signaling and transplantation tolerance (100). Xiong et al. reported that miR378 was decreased in renal allograft with IF/TA. Functionally, miR378 reduced IRI injury, inflammatory cell infiltration, and subsequent fibrosis in renal allografts (101). Moreover, miR-21, miR-210 and miR-4639 in plasma exosomes were significantly higher in CAD, and these 3 circulating exosomal miRNAs in combination may predict post-transplant renal graft dysfunction (102). It is worth noting that miR-21 upregulation in urine not only predicts DGF in the early phase post-transplantation as mentioned above (88), its overexpression in urine (103) and plasma exosomes (104) was associated with moderate to severe IF/TA and worse allograft function in the long term.

Very little is known about lncRNA in IF/TA following kidney transplantation. Nonetheless, 6 lncRNAs candidates (AC126763.1, RP11-280K24.1, LINC01137, WASIR2, RP1-276N6.2 and AD000684.2) were suggested to predict the development and progression of CAD using the GEO datasets of 407 biopsies from 3 different studies (118).

## Epigenetic Regulation in the Recipient on Kidney Graft Outcome

In addition to the epigenetic changes caused by the procedure of kidney transplantation, the condition of the recipients may also play an important role. Most of the recipients have had ESRD and a period of dialysis. The primary cause of ESRD and the dialysis vintage in the recipients may induce significant epigenetic alterations in these patients and, after transplantation, may also affect the outcome of the kidney graft. For example, diabetic kidney disease as a major complication of diabetes mellitus is associated with remarkable changes in epigenetics (121, 122). Much less is known about the epigenetic changes in dialysis. However, dialysis is known to induce a variety of changes ranging from those in gene expression to metabolism (123, 124). Therefore, the epigenetic status of the recipient, especially the primary cause of ESRD and prior dialysis, may affect the epigenetic regulation and outcome of the kidney graft.

## Conclusions and Perspectives

Epigenetic regulation plays a crucial role in renal pathophysiology and its emerging effect in kidney transplantation has been recognized in recent years. There are significant changes in DNA methylation and histone/non-histone acetylation in IRI of renal transplantation, although the underlying mechanisms remain to be clarified. Meanwhile, immune response is of vital importance in allo-response to the graft. The increasing knowledge of DNA methylation and acetylation of Foxp3 have shed new light on the mechanism and possible therapeutic targets. However, the immunological process in kidney transplantation is very complex and further investigation of epigenetic regulation is warranted. Additionally, microRNAs have been intensively studied for their regulation and their values in diagnosis and treatment of kidney dysfunction following transplantation. In contrast, the investigation of other non-coding RNAs is still emerging, and much less is known about their functions and regulation in kidney transplantation.

In the epigenetic mechanisms discussed, microRNA regulation is mostly close to clinical application in kidney transplantation, because the regulatory and predictive roles of microRNAs have been reported in quite a few preclinical studies and clinical trials. For DNA methylation, recent advances in computational tools can unravel DNA methylome in kidney transplant recipient that may lead to novel epigenetic biomarkers and therapies. In contrast, the investigation of histone modifications in kidney transplantation is still in its infancy and it is a long road from basic science to clinical translation. Nonetheless, specific pharmacological agents targeting histone modifications, for example HDAC inhibitors, may have therapeutic potentials in kidney transplantation.

Obviously, there are plenty to learn about the epigenetic mechanisms that contribute to various pathological processes in acute and chronic graft dysfunction following kidney transplantation. Further research in this area will provide opportunities for the discovery of new diagnostic biomarkers and the development of novel therapeutic strategies.

## Author Contributions

XX and ZD contributed to the conceptualization, design, and outline of this review. XX prepared the original draft with figures. XX, JZ, GD, and ZD revised and edited this review. All authors have read and agreed to the published version of the manuscript.

## Conflict of Interest

The authors declare that the research was conducted in the absence of any commercial or financial relationships that could be construed as a potential conflict of interest.

## Publisher’s Note

All claims expressed in this article are solely those of the authors and do not necessarily represent those of their affiliated organizations, or those of the publisher, the editors and the reviewers. Any product that may be evaluated in this article, or claim that may be made by its manufacturer, is not guaranteed or endorsed by the publisher.
